# Structural insights and shedding light on preferential interactions of dietary flavonoids with G-quadruplex DNA structures: A new horizon

**DOI:** 10.1016/j.heliyon.2023.e13959

**Published:** 2023-02-20

**Authors:** Sagar Bag, Mangal Deep Burman, Sudipta Bhowmik

**Affiliations:** a Department of Biophysics, Molecular Biology and Bioinformatics, University of Calcutta, 92, A.P.C. Road, Kolkata, 700009, India; b Mahatma Gandhi Medical Advanced Research Institute (MGMARI), Sri Balaji Vidyapeeth (Deemed to Be University), Pondy-Cuddalore Main Road, Pillayarkuppam, Pondicherry, 607402, India

**Keywords:** G-quadruplex, G-quadruplex ligands, G-quadruplex-DNA probes, Dietary flavonoids, G-quadruplex-dietary flavonoids interaction

## Abstract

G-quadruplex, a structurally unique structure in nucleic acids present all throughout the human genome, has sparked great attention in therapeutic investigations. Targeting G-quadruplex structure is a new strategy for the drug development. Flavonoids are found in almost all dietary plant-based beverages and food products; therefore, they are ingested in significant proportions through the human diet. Although synthetically developed drug molecules are used vigorously but they have various adverse effects. While on the other hand, nature supplies chemically unique scaffolds in the form of distinct dietary flavonoids that are easily accessible, less poisonous, and have higher bioavailability. Because of their great pharmacological effectiveness and minimal cytotoxicity, such low molecular weight compounds are feasible alternatives to synthetic therapeutic medicines. Therefore, from a drug-development point of view, investigation on screening the binding capabilities of quadruplex-interactive small natural compounds like dietary flavonoids are expected to be highly effective, with a particular emphasis on the selectivity towards polymorphic G-quadruplex structures. In this respect, quadruplexes have scintillated research into their potential interaction with these dietary flavonoids. The purpose of this review is to offer an up-to-date close-up look at the research on their interaction with structurally varied dietary flavonoids with the goal of providing newer perspectives to construct novel therapeutic agents for next-generation disease managements.

## Highlights

• Humans consume considerable amounts of dietary flavonoids through their diet, for making them viable substitutes for conventional medicinal drugs.
• Functional characterization and novel interactions between different types of dietary flavonoids with G-quadruplexes has been explored.
• Interactions between G-quadruplex DNA structures and dietary flavonoids studied as potential molecular targets for prospective disease control.
• Enlighten the structural variation between dietary flavonoids with respect to their binding to G-quadruplex DNA structures for providing knowledge that can be used to develop novel drugs for next-generation disease managements.

## Graphical abstract

**Figure:**
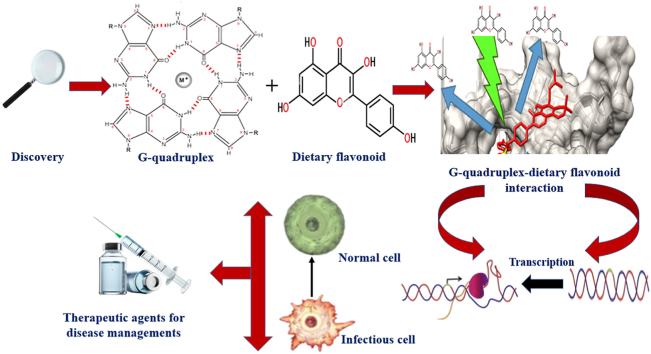

## 1 Introduction

**Fig. 1:**
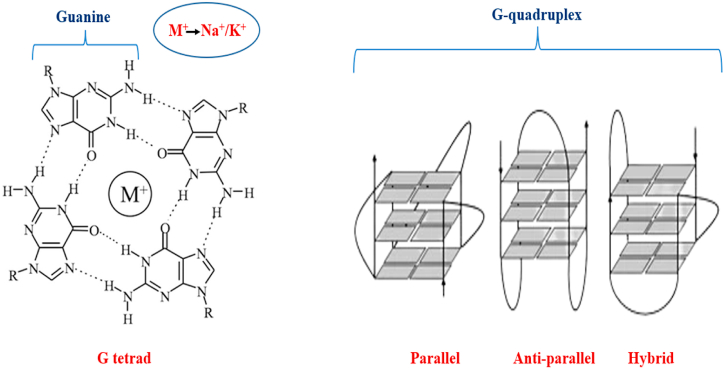
G-quadruplex schematic layouts. G-quadruplex structures in this picture depict three stacks of G-tetrads and are divided into three categories based on their topology: parallel, antiparallel, and hybrid (direction of the 4 strands). M^+^ and dots in the G-tetrad represent cationic ions, Na^+^ or K^+^, and Hoogsteen hydrogen bonding, accordingly.

G-quadruplex, a structurally unique structure in nucleic acids present across the human genomes, has piqued the curiosity of many researchers in the arena of therapeutic investigation. G-quadruplex structure, which is found in important regulatory areas of oncogenes, affects translation, splicing, transcription, telomere integrity, and other processes [1,2]. Alteration in its integrity and architecture cause oncogenes to express differently, resulting in cancer. Therefore, targeting G-quadruplex architectures with small compounds biologics has attracted the attention of researchers. G-quadruplex structures can occur in regions with longer loops or fewer than three guanines per repetition, and also in areas that do not adhere to this strict G-quadruplex pattern. The stability of the G-quadruplex structure is determined by a variety of parameters, including the number of guanines per repetition and the loop length [3]. Human DNA contains over 50% repetitive DNA sequences; whenever these sequences unwind throughout processes such as transcription or replication, they transform into single strand lengths that can wrap into a wide range of non-canonical forms. This contains G-quadruplex DNA structures, i-motifs, cruciform DNA, triplex, and other similar structures. Secondary constructs created by guanine-rich DNA that may form or adopt a G-quadruplex structure, but it cannot generate it in the presence of monovalent metallic ions (Fig. 1). While G-quadruplex was initially found *in vitro*, growing evidence suggests that this unusual nucleic acid construct is also generated in living cells on a genome-wide scale [[4], [5], [6]]. These guanine-rich repetitive sequences are likely to stack on one another to form guanine planes termed G-tetrads. Within a quadruplex, G-tetrads are stacked one on top of each other, held together by phi-phi non-bonded attractive interactions. They are further driven by presence of Na^+^/k^+^ (monovalent cations). To construct such tetrads, the DNA threads must have a distinctive folding geometric feature than the conventional B-forms, and they have been demonstrated to produce unique hydrogen-bonded base pairs designated as Hoogsteen bonds (Fig. 1).

G-quadruplex DNA structures are widely categorised into three primary varieties based on strand polarisation: “anti-parallel” (couple of the four strands pointed in the same orientation), “parallel” (all strands pointed in the same orientation), and "hybrids'' or “mixed parallel-antiparallel” (three of the strands run in the same orientation, whereas the fourth runs in the opposite way) (Fig. 1) [7]. A G-quadruplex architecture is stabilised by a set of attributes, including base stacking, hydration structure, hydrogen bonding, and electrostatic attraction. Because DNA bases are non-polar in composition, they stack atop one another to decrease the surface accessible to polar solvent. They are then stabilised by electrostatic, hydrophobic and van der Waal forces. Furthermore, both non-telomeric as well as telomeric quadruplexes are linked by a diverse set of proteins that either stabilise or destabilise them. All of these interactions, however, are insufficient to maintain the G-quadruplex structures [8]. As possible therapeutic candidates for treatment of cancer, two major groups of G-quadruplex DNA structures have garnered attention: (A) They are capable of formation at the single-stranded guanine-overhang of telomeric DNA, and (B) those inside genomes, particularly within the promoter regions of specific oncogenes like c-MYC, VEGF, c-KIT, PDGF-A, k-RAS, Hsp90 and HIF1 etc [7]. It has recently been suggested that ligands which preferentially attach G-quadruplex DNAs can down-regulate their over-expression, most probably owing to polymerase pausing at the site of G-quadruplex structure, or result in modified protein expression, and may also stimulate DNA damage reactions, thereby amplifying their anticancer properties [9]. Furthermore, targeting certain higher degree secondary isoforms gives a better degree of specificity and selectivity than that of other typical cancer therapeutic targets.

Generally, cellular DNA exists in the B-form, with the regular H-bonding as seen in the Watson-Crick DNA model. Alternative nucleic acid structure that differs from double stranded structure have essential biological functions. G-quadruplexes are common in the human genome, and also in viruses, bacteria and plants. The distribution of G-quadruplexes shows regulatory functions in a variety of cellular activities such as recombination, replication, transcription, translation, and telomere preservation. Furthermore, the polymorphism of G quadruplexes makes it difficult to interpret and design particular recognition of appropriate structure by ligands. The polymorphism results from alterations in strand stoichiometry, location and orientation of the loops linking guanines, and reciprocal orientations of G-tracts in the structure's interior [10]. In human genomes, G quadruplex-DNA forming sequences frequently found near, and are enriched at chromosomal mutation hotspot regions in disease-associated genes, connecting them in genomic instability and diseases. Recognizing G-quadruplex’ biological importance has stimulated the discovery and production of ligands that bind with G-quadruplex DNA structures and modulate their conformation and functionality. Despite some important advances in the area, the key concern remains the trade-off between selectivity and affinity, which might be addressed with a complete characterization of G-quadruplex/ligand associations. Because the G-quadruplex structure is destabilised in most diseases like cancers [8], discovering ligands that particularly stabilise the G-quadruplex formations is a prospective research field in chemistry and biology [9]. The capacity of smaller compounds to stabilise G-quadruplex DNA and impede with telomere elongation in cancer cells by blocking the enzyme telomerase has emphasised the potential importance of quadruplexes for anti-cancer therapeutic targets. Because of their physiologically significant functions in oncology, G-quadruplex DNAs have emerged as an attractive target for therapeutic discovery. Several small compounds that bind and stabilise G-quadruplex structures have been investigated to date. The majority of these compounds have a vast surface area and attach to G-quadruplex DNA through π- π stacking interaction.

Generally, naturally available plant chemicals have been shown to be extremely effective anti-cancer drugs that are far less toxic than synthesized derivatives, and they are commonly utilized in a variety of pharmacological functions with greater biological benefits. Flavonoids are polyphenolic substances that naturally occur in medicinal plants, vegetables, fruits, flowers, and a range of beverages (coffee, wine, fruit juice, and tea) [11,12]. Flavonoids have anti-viral, anti-inflammatory, antioxidant, anti-tumour properties due to free radical absorption and complex formation with metal ions. These health-promoting actions may include the suppression of human body enzymes. Multiple findings indicate that these dietary flavonoids can serve as antioxidants by mitigating DNA damage and scavenging reactive oxygen species, preventing DNA adduct generation, promoting DNA repair, interfering with chemical damage via inducing changing signal transduction pathways [13]. The clinical use of flavonoid-rich foods provides unique opportunity to investigate the function of dietary flavonoids in disease prevention and management. There is currently no agreement on standardised dosages or sources of flavonoids for clinical trials. Moreover, the association of natural flavonoid substances with biomolecules (DNA, protein, RNA) can maintain the non-covalent connection via π–π stacking interaction [14,15]. Daidzein, rutin, quercetin and genistein have been shown to interact with G-quadruplex DNA, despite the fact that their non-planar variants are quite distinct from those of typical G-quadruplex ligands with cross-linked aromatic rings. This generated our interest in researching how flavonoids recognise G-quadruplex DNA structures. The discovery of dietary flavonoids as G-quadruplex binding ligands that can stabilise the structure might be viewed as an innovative and potential technique for efficiently preventing diseases and overcoming drug resistance issues. The primary goal of this study is to gather information that how these naturally occurring dietary flavonoids interact with the G-quadruplex DNA and how these dietary flavonoids-G-quadruplex DNA interaction helps to construct advanced therapeutic strategies for next-generation disease managements.

Based on the existing research, in this review, we highlighted and explored the interactions between several types of dietary flavonoids with G-quadruplexes. This systematic review demonstrates that the structural variations between dietary flavonoids cause these chemicals to interact differently with distinct DNA structures. Furthermore, the goal of this review is to provide a structural underpinning for the association and maintenance of the G-quadruplexes by the most prevalent naturally occurring dietary flavonoids. This study will serve as a solid foundation for future research and the production of safer, more effective medications for a wide range of diseases.

## 2 *In vivo* organization and functional characterization of the G-quadruplex

According to a bioinformatics study, the human genome contains 700,000 potential G-quadruplex producing sequences [16]. The fascinating fact is that such constructions are not distributed randomly; somewhat more, they are strongly associated with major regulatory areas such as telomeres, replication origins, promoters, 3′-UTR and 5′-UTR, RBS (Ribosome Binding Sites), and long non-coding RNA in which they attenuate expression of genes, implying that G-quadruplex structures may play an important role in the regulation of a wide range of cellular procedures. Quadruplexes found in the promoter often suppress gene expression, but those found in the 5′-UTR and 3′-UTR generally influence miRNA binding, alternative polyadenylation, pre-mRNA splicing, translation, and mRNA targeting [17]. Surprisingly, such structures are frequently found in proto-oncogenes but appear to be lacking in tumour suppressor genes, implying that G-quadruplex structures have been selected for evolutionary reasons depending on their functionality [[18], [19], [20]].

**Fig. 2:**
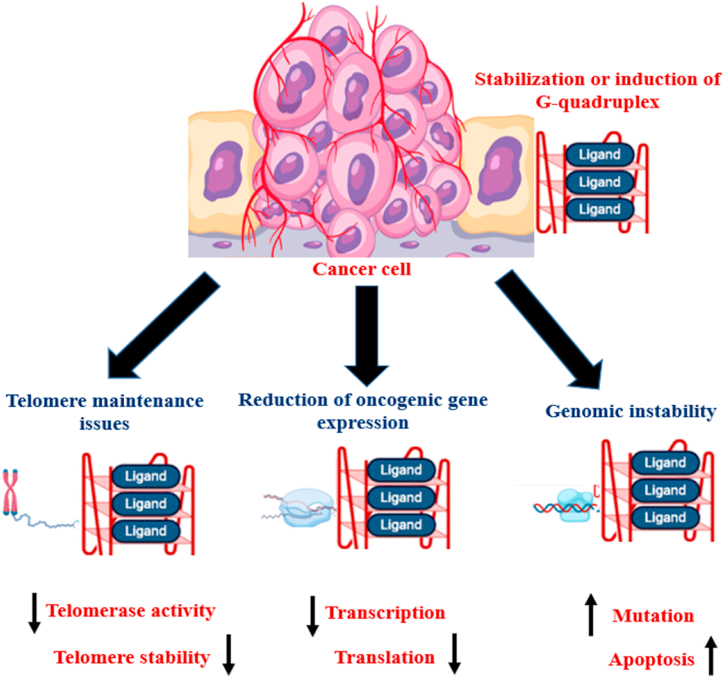
Schematic diagram depicting the impact of G-quadruplex ligands on cancerous cells. The majority of G-quadruplex ligands promote slower development. These growth alterations are the result of changes in biological mechanisms. Based on the ligands and cell types G-quadruplex stabilisation can result in alterations in (a) telomere maintenance (b) oncogenic gene expression (c) enhanced genomic instability.

G-quadruplex forming sequences were discovered in telomeric DNA of humans for the first time. The stability as well as structure of telomeres are linked to aging, cancer, and genetic stability. G-quadruplex works as a tumour suppressor in telomeres by suppressing telomerase and so preserving the telomere (Fig. 2). Telomeric quadruplexes are substantially conserved and are linked to a variety of telomere associated proteins [8,21]. Several G-quadruplex structures are also essential to replication commencement. According to Valton and his colleagues, removal of G-quadruplex motifs in the med14 origins resulted in a severe drop in origin of replication activity. Additionally, point mutation in the conventional G-quadruplex motif β^A^ resulted in much reduced replication origination performance as compared to wild type cells [22].

*In-silico* investigations revealed that G-quadruplex motifs are typically found in the promoter sequences of over 20,000 genes [23]. A potentially persistent G-quadruplex structure is seen in numerous oncogenes, including c-MYC, c-KIT, hTERT, K-RAS, VEGF, BCL2, PDGF-A, and others [24]. In most of these circumstances, the G-quadruplex forming sequences works as a transcriptional repressor, retaining a threshold amount of gene transcript. According to research, stability of the G-quadruplex architecture in NHEIII1 leads to a reduction in c-MYC transcription [25]. Likewise, the guanine rich strands on the VEGF gene's proximal promoter region governs its production. The stabilisation of this G-quadruplex reduces VEGF expression. Several cell regulatory networks and the quadruplex in the non-template strands precisely govern ribosome synthesis in the nucleolus. The speed limiting stage in ribosomal synthesis is rRNA biosynthesis. The ribosomal DNA (rDNA) contains two G-quadruplex structures, NUC 19P and NUC 23P, that regulate the speed of rRNA synthesis. The synthesis and stability of these quadruplexes by nucleolin proteins causes rRNA upregulation. In this case, quadruplex serves as a transcriptional stimulator [8,26].

There are numerous more instances that support the notion that G-quadruplexes are important regulatory gatekeepers inside the cell. A genome-wide assessment of G-quadruplex motifs in numerous species found that they are evolutionarily conserved, confirming their crucial functional significance in biology. Targeted antibodies towards G-quadruplexes in the telomeric DNA of the ciliate *Stylonychia lemnae* provided direct indication for the presence of G-quadruplexes at telomere regions. The prescriptive and quantifiable visualisation of DNA G-quadruplex structure in mammalian cells has been recently discovered. The G-quadruplex was detected in telomere regions and outside of the telomeres using the structure-specific antibody BG4, and it generated in a replication-dependent way during the cell cycle [27]. More crucially, after treatment with the G-quadruplex attaching ligand, the frequency of BG4 foci expanded, demonstrating that the small molecules could capture and stabilise a G-quadruplex in mammalian cells. Furthermore, effective high DNA sequencing revealed that G-quadruplex structures co-localized with antibodies BG440, H2AX43, 1H641, hf242, and the DEAH/RHA family of helicases [[28], [29], [30]]. All this compelling irrefutable evidence not only supports the presence and position of G-quadruplex structures in the genome, but also indicates the important role of G-quadruplexes. A particular small chemical might modify these structures in cells, indicating that targeting a G-quadruplex has therapeutic promise.

## 3 G-quadruplex–ligand: mode of interaction

The prevalence of G-quadruplex within and around important oncogenes, as seen above, gives it a promising location for therapeutic targeting. Numerous ligands attach to G-quadruplex, either stabilising or destabilising it (Fig. 2). Terminal stacking is the preferred way of coupling since it does not necessitate the transitory unstacking of G-tetrads. Many ligands, instead of attaching to pre-formed quadruplexes, aid in the folding of the guanine-rich regions to quadruplex. Kinetic research has improved our knowledge of how these processes occur. One such chemical is PIPER, a perylene derivative that facilitates the folding of the telomeric sequences 5′TTAGGG3′ to tetrameric and dimeric G-quadruplex structures [31]. Many ligands share common structural and chemical features, which is a planar aromatic circle with a charged lateral chain. The G-quadruplex provides a good substrate for ligand binding via π-π stacking. Several attach to the G-quadruplex by exploiting the groove produced by the backbone network, which primarily have included the terminal G-tetrads, the intermediate G-tetrads, the loops/backbones/grooves, and the middle channels [32,33]. Planar heterocyclic ligands engage with G-quadruplexes mostly by π-π mounting on the terminal G-tetrads and less often by intercalating into the G-tetrads [34]. Ligands having amino moieties can engage with the G-quadruplex’ grooves and negative charged phosphate framework [35]. Methylation or protonation positively charges the amino groups, which might also result in improved identification and tighter binding to the groove as well as to the negatively energized phosphate backbone via electrostatic associations [3]. The side chains may detect the grooves loop bases by making hydrogen bonds that increase the ligands' solubility in water. More intriguing, small-sized ligands with lengthy side chains that are positively charged, can occupy the core channel of a G-quadruplex, increasing the stability of quadruplexes [36]. According to research, whereas planar ligands have the capability to insert into the G-tetrad layers, charged compounds are more likely to engage with the loops, backbones or grooves of G-quadruplex complexes [37,38]. In a recent investigation, it has been found that, in potassium chloride solution, phen-DC3, among the most notable G-quadruplex ligands in terms of strong ligand binding and specificity, induces dTAGGG (5′TTAGGG3′)_3_ to completely alter its fold from a hybrid-1 to an antiparallel chair-type configuration, in which the ligand intercalates between a 2-tetrad unit and a pseudo-tetrad unit, expelling one potassium ion. This exceptional high-resolution NMR (Nuclear magnetic resonance) structure reveals for the very first time a genuine ligand complexation into an intramolecular G-quadruplex [10].

## 4 G-quadruplex-DNA probes and biological outcomes

Ligand-mediated structural regulation of G-quadruplex-DNA in promoter sequences of genes might be a strategy for controlling detrimental gene expression. Investigations to increase G-quadruplex-DNA production and stability have revealed the possibility for transcriptional regulation, perhaps downregulation or upregulation of expression of genes, based on G-quadruplex-DNA associations with transcription-regulating proteins. There are several reviews accessible on ligand targets of promoter-specific G-quadruplex-DNA production. G-quadruplex-DNA structural destabilisation, on the other side, is a largely unexplored method of controlling gene expression. This was demonstrated by the enhancement in translation effectiveness of (CGG)_99_ firefly luciferase mRNA *in vitro* after exposure with the TMPyP4 ligand [39].

**Fig. 3:**
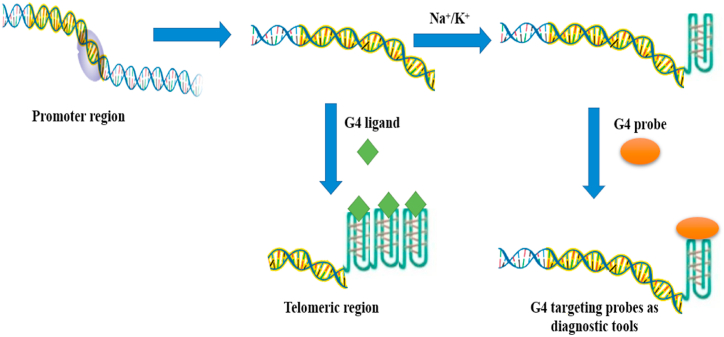
Schematic representation of G-quadruplex (G4) targeting probes and G-quadruplex stabilising ligands.

Through chemical fluorophores to metallic complexes, the number of described G-quadruplex-DNA-stabilising chemicals offers ligands that can be employed as possible probes of G-quadruplex-DNA conformation (Fig. 3). In parallel to the “light up” as well as “light off” probes that display a rise or fall in fluorescent/spectroscopic indicators following G-quadruplex-DNA interacting, high-affinity ligands ‘labelled’ with fluorophores have been explored (Fig. 3). Several ligands with potential biophysical properties have been investigated in cells using chromosomal imaging [10,37]. While preferential staining in the nucleoli/nucleus has been found in a few experiments, the findings may not be taken as the presence of G-quadruplex-DNA complexes *in vivo* because of non-specific adherence to other proteins [40,41]. Furthermore, the cellular localisation of several ligands varies based upon when the cells were alive or fixed, as well as the form of fixation or crosslinking utilized. Employing live cells, orthogonal tests like pulldown, sequencing crosslinking can validate such labelling as an actual observation of G-quadruplex-DNA structures.

Throughout the last decade, significant progress has been achieved in the discovery of G-quadruplex-specific compounds effective for secondary structure stabilisation [42,43]. The search for quadruplex-selective ligands has progressed from the early problem of identifying quadruplexes from canonical DNA to the current difficulty of differentiating quadruplex structures from one another. Not unexpectedly, fluorescent specific ligands able to recognise quadruplex complexes *in vivo* have encountered similar difficulties. There are many G-quadruplex-selective fluorescent probes that depend on an end-stacking detection process. These probes' recurring motifs comprise heterocyclic compounds like benzothiazole, the retention of charged centres, and prolonged conjugation. Given the significantly restricted collection of G-quadruplex-selective fluorescent agonists, it is possible that such drugs might have a dual theranostic function, allowing for more confirmation of G-quadruplex-targets in clinical situations.

## 5 What are dietary flavonoids?

**Fig. 4:**
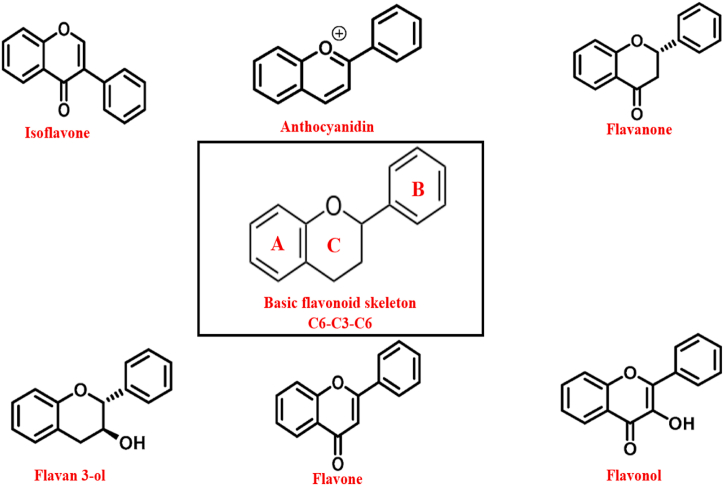
Fundamental chemical structure of flavonoids and their sub-groups.

Human health and physiology have been positively impacted by flavonoids. Research concentrating on the pharmacodynamic, and physiological actions of flavonoids have increased because of the recent discovery of several flavonoids with a variety of activities. Plant vacuoles contain secondary compounds called flavonoids. Flavonoids are naturally existing polyphenolic substances present in many vegetables, citrus fruits, seeds, and so on, and are thus absorbed in significant quantities into the human diet [44]. The number of flavonoids identified in the literature is over 10,000, ranking them third among the most prevalent bioactive substances in plants. Flavonoids are classified into isoflavonoids, anthocyanidins, flavones, flavanones, catechins, and flavonols [45] (Fig. 4). The primary roles of flavonoids in plants are to protect them from UV rays and infections as well as to aid in pollination by attracting pollinators. The two aromatic rings A and B, which are joined by the C ring, which contains three carbon atoms, are consisting of fifteen carbon atoms (C6–C3–C6) in the fundamental chemical composition of flavonoids (Fig. 4) [46]. Several investigations have revealed that flavonoids may defend against a variety of illnesses, including heart disease, cancer, neurological disease, as well as other age-related maladies. Flavonoids’ strong pharmacological efficacy and minimal cytotoxicity make them attractive substitutes to traditional medicinal medicines [[47], [48], [49]].

The worldwide variation in the number and varieties of flavonoids ingested Is determined by differences in lifestyle, gender, dietary preferences, and socioeconomic position, all of which led to disparities in disease prevalence when combined with environmental variables [13]. Estimating total flavonoid consumption is challenging due to the scarcity of food content information. It is believed that people consume a few hundreds of milligrams every day. In most potential epidemiological investigations, consumption of flavones and flavonols was shown to be inversely related to future cardiovascular disease. Just one prospective trial demonstrated flavonols to be cancer protective. Since flavonoids in foods are coupled to sugars as beta-glycosides, these compounds were thought to be non-absorbable. The sugar moiety influences the bioavailability and absorption of these dietary flavonoids [50]. Dietary flavonoids’ interaction with G-quadruplex DNAs have received relatively little attention. However, the interaction of dietary flavonoids with G-quadruplex DNAs must be studied further to offer information on ligand-target stoichiometry, selectivity for G-quadruplex DNAs over double stranded DNA, and the capacity to stabilise the quadruplex structure.

## 6 Interaction of G-quadruplex DNAs with dietary flavonoids

Flavonoids have several positive benefits, ranging from control of different enzymatic activities to prevention of cellular proliferation, although the underlying processes are not entirely known. Increasing data suggests that flavonoids may target G-quadruplex structures [51,52]. Luteolin, rutin, quercetin, genistein, kaempferol, hesperidin, myricetin, and daidzein are prevalent dietary flavonoids that have garnered substantial interest for its anti-metastatic, anti-angiogenesis and anti-proliferative activities [53,54]. Previously, one study found that quercetin interacts with monomeric and dimeric G-quadruplex structures generated by a short repetition of the human telomeric region. Yang et al., in 2013 evaluated the associations of the c-myc G-quadruplex complex with a variety of pyridinium side groups comprising flavone derivative products [55]. They discovered that these chemicals had a higher affinity for c-myc G-quadruplex DNA than for other quadruplexes and duplexes [56].

**Fig. 5:**
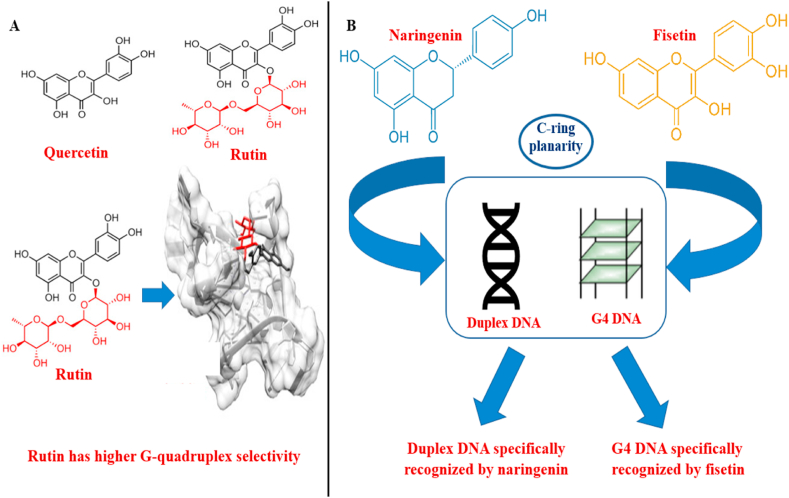
Interaction of G-quadruplex with dietary flavonoids. (A) Rutin, a flavonoid glycoside, has higher G-quadruplex selectivity than quercetin. The rutinose portion is highlighted in red. Rutin was expected to interact through stacking, the most effective strategy for G-quadruplex stabilisation, in this computational model. (B) The structural differences between naringenin and fisetin allow these compounds to interact differently with various DNA configurations. C-ring planarity seems to be a critical element in preferred G-quadruplex DNA binding of flavonoids. (For interpretation of the references to color in this figure legend, the reader is referred to the Web version of this article.)

The most prevalent dietary plant flavonoid, quercetin, has been discovered to have various advantageous benefits on human health [57]. Furthermore, quercetin has a high spectroscopic curiosity due to its distinctive ‘two colour’ fluorescence characteristics, which may be used to detect G-quadruplex-DNA. In aqueous solution containing, free quercetin emits little fluorescence. Moreover, when G-quadruplex-DNA as well as other target biological molecules are bound, significant fluorescence signals are detected. According to our research, quercetin preferentially recognises VEGF promoter G-quadruplex-DNA over the other sequences of G-quadruplex-DNA and duplex DNA investigated. Quercetin's preferred association with VEGF G-quadruplex-DNA is demonstrated by a notably significant rise in time-resolved and steady-state fluorescence intensity characteristics, in comparison to other G-quadruplex-DNA structures and duplex DNA, in which only minor changes are seen. Furthermore, the establishment of a G-quadruplex-DNA-based logic gate detecting framework with quercetin and VEGF G-quadruplex-DNA in diverse pH settings is a unique utilisation quercetin, which is distinguished by its fascinating and extremely sensitive fluorescence [58]. In an investigation, the quantum chemical analysis was utilized to analyse the behaviour of the quercetin in interactions with DNA tetrads. The findings shows that the quercetin interacts effectively with the GCGC tetrad, has excellent structural strength, and has a greater interaction energy. The quantum chemistry discovery prompted researchers to use molecular dynamic simulation to further examine the quercetin with G-quadruplex DNA (c-myc oncogene). In the experiment, several environmental variables (temperature and pH) are employed. Quercetin binds with the 5′ and 3′ terminals of G-quadruplex DNA. These observations assist us understand the binding properties of quercetin and its anti-cancer cell activities [14]. Quercetin stacks and stabilises the Pu24T G-quadruplex architecture at the 5′ and 3′ G-tetrads through π -π stacking engagements. *In vitro* experiments on HeLa cells have shown that quercetin causes apoptosis-mediated cellular death and decreases gene expression of c-myc. This investigation highlights flavonoids' potential as a prospective option for hitting the c-myc promoter area and therefore serving as a possible anti-cancer drug [56]. Researchers computed the bonding energies of quercetin binding positions with polymorphic telomeric G-quadruplex DNA architectures (anti-parallel, parallel, and mixed) in a study. They also looked at quercetin's ability to attach to RNA G-quadruplex structures and cancer proto-oncogenes The quercetin's binding energies determined independently for each G-quadruplex structure suggest that quercetin might be employed as a pioneer molecule to target polymorphic telomeric G-quadruplex structures as well as cancer proto-oncogenes, making it a viable natural medicinal molecule for anti-cancer treatments [15]. In a recent work, the interactions of rutin, a glycosidic derivative of quercetin isolated from *Styphnolobium japonicum* (L.), with G-quadruplex DNA was studied using molecular docking and ESI-MS. While rutin and quercetin had comparable G-quadruplex binding propensity values, rutin was distinguished by its improved preference for G-quadruplex over ds DNA. Furthermore, CID (Collision-Induced Dissociation) experiments revealed that rutin more effectively stabilises the G-quadruplex structure, and molecular docking suggested stacking as the preferable interaction type [59]. Rutin was expected to interact through stacking, the most effective strategy for G-quadruplex stabilisation, in computational model (Fig. 5A).

Naringenin and fisetin are dietary flavonoids found in citrus fruits (apple, grape, onion, strawberry, coffee, and tea). Naringenin could execute several biological processes in order to cure a wide variety of inflammatory disorders and pathological ailments caused by oxidative stress. Naringenin's immunomodulatory properties have recently been studied as a viable alternative therapy for autoimmune illnesses [60,61]. Fisetin has already been discovered to have anti-apoptotic, anti-angiogenic antioxidant and anti-tumorigenic properties [62]. Researchers performed a combo of spectroscopic as well as computational experiments to investigate the biological interactions of two structurally related compounds (selected as examples of two separate groups of dietary bioflavonoids) with the parallel G-quadruplex and the duplex DNA. Our studies revealed that the structural variations between naringenin and fisetin enable these chemicals to interact differently with distinct DNA structures. Fisetin binds more strongly with parallel G-quadruplex structures than that of the duplex DNA, while naringenin has a higher affinity for duplex DNA than for parallel G-quadruplex DNA. Docking study's findings reinforce the spectroscopic findings, and it was confirmed that these ligands are outwardly stacked in the G-quadruplex DNA structure. The flavonoid structure's C-ring planarity seems to be a critical element in preferred G-quadruplex DNA binding of flavonoids. This study also highlights the use of flavonoids' dual fluorescence characteristic to explore specific G-quadruplex DNA binding and sets the groundwork for future implications in this area (Fig. 5B) [63]. It may be worthwhile to investigate the potential of fisetin's precise ‘two colour’ fluorescence (as well as other fluorescent flavonoids) as a potential approach for the identification and quantification of G-quadruplex DNA, for example, in human cancer cells.

Researchers used spectroscopic as well as docking experiments to investigate the affinities of the structurally similar plant flavonols morin and kaempferol with distinct duplex DNA and G-quadruplex-DNA sequences (c-MYC, h-TELO, c-KIT1, VEGF and c-KIT2). The findings suggest that kaempferol interacts preferentially with VEGF G-quadruplex-DNA over duplex DNA and other G-quadruplex sequences. Furthermore, kaempferol improves the thermal durability of VEGF G-quadruplex-DNA. Morin, on the other hand, has a significantly lesser amount of affinity with both different G-quadruplex-DNAs and duplex DNA, with no notable structural specialization. The differing DNA binding behaviours point to a critical role for the 2′-OH functional group in the flavonol moiety's B-ring. While kaempferol has a comparatively planar shape, morin has a considerably non-planar configuration due to steric hindrance from the extra 2′-OH group. This little structural change appears to be critical for kaempferol and morin's capacity to engage with VEGF G-quadruplex-DNA. Therefore, kaempferol (but not morin) appears to be an efficient ligand for VEGF G-quadruplex-DNA, suggesting that it might be used to regulate gene transcription in cancer cells [64].

Quercetin, a dietary flavonoid, known to interact with the Tel7 G-quadruplex sequences d- (TTAGGGT). A study has been performed to focus on the interaction of 4 distinct flavonoids, quercetin, genistein, rutin, and, luteolin, with the Tel7 G-quadruplex sequences. To explore the binding mechanism of these dietary flavonoids to Tel7 G-quadruplex DNA, NMR experiments with additional biophysical methods including as uv–visible absorption, steady-state & time-resolved fluorescence spectroscopy, circular dichroism were studied. According to the findings, all of the flavonoids interact to Tel7 G-quadruplex DNA. Quercetin also binds to Tel7 G-quadruplex DNA by intercalation between both the G6pT7 and T1pT2 stages, according to comprehensive structural investigations [65]. In an experiment, the interaction of dietary flavonoid myricetin with telomeric G-quadruplex has been investigated. In this study, it has been found that myricetin inhibited telomerase activity and significantly reduced the expression of hTERT. Low resolution spectroscopic techniques in combination with NMR spectroscopy as well as Molecular Dynamics Simulation (MDS) have been employed to uncover the mechanistic mechanisms of myricetin's interaction with H24. ITC (Isothermal Titration Calorimetry) was performed to determine the thermodynamic viability of myricetin-H24 complex production. ITC findings indicate thermodynamic characteristics of myricetin-H24 complex production and assign a 1:1 binding stoichiometry. NMR studies and simulation studies show that myricetin preferentially binds to H24 via the 3′ terminus of the G-quadruplex [66]. The interaction of dietary flavonoids with G-quadruplex DNA structures, on the other hand, has to be thoroughly researched in order to give detailed data on ligand-target stoichiometry, preference for G-quadruplex over duplex DNA, and the capacity to stabilise the G-quadruplex structure.

Beside flavonoids, there are also other dietary compounds that are found to interact with G-quadruplex DNA structures. A biological active substance Curcumin (turmeric) is a hydrophobic phenolic compound derived from the rhizome of *Curcuma longa* which may function as a G-quadruplex binding agent. Curcumin's G-quadruplex binding affinity was explored using structural characteristics shared through other effective G-quadruplex binder compounds, such as the electron charged aromatic surface offered by curcumin's alkoxy-side extremities, and the flat aromatic rings that are stuck by an intrinsic hydrogen bonding crosslink. Curcumin comprises 2 aromatic ring configurations with o-methoxy phenolic groups which are linked by a 7-carbon coupler made up of an alpha, beta-unsaturated beta-diketone molecule. Curcumin has an effective electron transfer capacity owing to its unusual structure and many functional units, which include a beta-diketone and multiple π-electrons capable of conjugating between 2 phenyl rings [67,68]. Curcumin has previously been discovered to interact with the telomeric G-quadruplex during molecular crowding conditions. In a recent study, a group of researchers investigated the impact of curcumin's association with the c-MYC G-quadruplex. Several biophysical and in silico experiments have been conducted to investigate the binding characteristic of the curcumin-c-MYC-quadruplex. This study demonstrates that curcumin can stabilise and maintain the parallel configuration of the indigenous c-MYC-quadruplex sequences. Curcumin interacts to the 3′ termini of the c-MYC quadruplex to develop a stable complex, according to the findings of molecular docking and MD modelling experiments [69]. Curcumin's binding affinities and specificity to c-MYC G-quadruplex confirms curcumin as a viable natural product to be exploited as a drug-model to build G-quadruplex targeted ligands.

Gallic acid (GA) is a phenolic compound found in a variety of dietary sources, including many vegetables and fruits. GA is more important than other phenols because of its powerful and specific antitumoral activity in colorectal cancer [70,71]. The differences in effectiveness appear to be attributable to differences in chemical makeup. In fact, substances having a higher quantity of hydroxylic groups outperformed those with a smaller number in terms of anticancer efficacy. GA, which contains 3 hydroxyl groups connected to 3, 4, and 5 positions of a benzoic acid centre, has been found to be more efficient than that of other phenols in this respect. GA's activity as a ligand of DNA G-quadruplex structures, which explains some of its antitumor actions, particularly transcriptional suppression of c-MYC and ribosomal genes. Furthermore, GA shared certain activities with other known G-quadruplex ligands, including such nucleolar stress, cell cycle arrest and induction of DNA damages. Applying a colorectal cancer xenograft model, researchers verified the antitumoral and G-quadruplex-stabilising effects of GA in an experiment. Furthermore, they demonstrate simply that GA might be investigated as a therapeutic drug in a patient group with colorectal cancer. This study shows that GA, a naturally occurring bioactive molecule in the diet, impacts gene expression by interacting with G-quadruplex structures both *in vitro* as well as *in vivo*, paving the way for G-quadruplex structure targeting phenolics (72). Nutrigenomics is concerned with the current molecular connections between genes and nutrition. Researchers discovered how a natural biologically active molecule found in our food, gallic acid, might influence gene expression via interactions with G-quadruplex structures. Investigations with cells *in vitro* and, more crucially, animals have deduced the stabilising qualities of gallic acid, which might be generalised to humans [72,73]. Gallic acid is intimately implicated in nutrigenomics, that ultimately determines people's health and illness, via this fundamental process.

The spectroscopic investigations on dietary flavonoid-G-quadruplex DNA interactions presented here provide a potent strategy for investigating their DNA binding by using the flavonoids' very reactive intrinsic fluorescence capabilities as their individual “reporter” for their associations with macromolecular targets. Therefore, a potential strategy for controlling the expression of different genes may be illustrated by the unique binding of certain dietary flavonoids to the G-quadruplex DNA structures.

## 7 G-quadruplex and dietary flavonoids interaction: advantageous or disadvantageous?

G-quadruplex, which regulates gene expression, has emerged as an attractive anti-cancer target. Because quadruplexes serve a variety of biological functions, targeting them has emerged as a popular field of drug discovery study. A new viewpoint on these motifs has resulted in the discovery of ligand compounds that engage with them. We've seen in this study that various dietary flavonoids have been designed to target G-quadruplex structures. In reality, the majority of these dietary flavonoids are unable to differentiate between distinct quadruplexes and exhibit preference to more than one G-quadruplex. Because G-quadruplex structures have a high polymorphism and dynamic shape, designing ligands that can recognise and react with these entities is tough. The main disadvantage of using such compounds as quadruplex targeting drugs is their off-target action and toxicity. These compounds can break down and create hazardous metabolites, resulting in poor body clearance. All these disadvantages must be resolved to produce a molecule as a medication. Any compound that wants to be a G-quadruplex targeted drug must meet the following criteria:

(I) It must be able to tell the difference between quadruplex and duplex and adhere to it.
(II) It must be able to distinguish between distinct G-Quadruplex structures.
(III) It ought to have a minimal off-target impact.
(IV) It must be cell permeable.
(V) In the cell, it must not break down into a harmful metabolite.

A molecule that meets all of these qualities has yet to be identified. Despite the availability of various anti-cancer drugs, the multi-drug resistant characteristic of cancer posed a challenge to traditional treatment. As a result, drug research necessitates the development of an alternate therapy, which is why recombinant compounds and peptides as drugs are thriving. Investigators are gradually concentrating more on developing G-quadruplex stabilising peptides. The current multimodal chemistry and phage display libraries approach enables one to analyse several peptides against a target and choose the best from lots all at once. Because peptides can fold back and forth on their own, they can adapt to changing G-quadruplex landscapes while attaching to them, enabling increased specificity and tighter adherence.

The unfortunate disadvantages that make G-quadruplex a difficult target can be addressed by merging computational and bioinformatics approaches, which can lead to the identification and creation of more new ligands capable of distinguishing various topologies of G-quadruplex with much greater accuracy. Using structure-based drug discovery and virtual database-based ligand screenings is the next stage in obtaining ligands that are specific to a quadruplex [74]. The X-ray crystal structures, and NMR of the G-quadruplex-dietary flavonoids combination have revealed insight on the atomic interconnections that occur among them, and this expertise may be used to build more effective drugs. Recent research and the development of new methodologies are extracting a plethora of information about G-quadruplex architectures, and it will not be long before we identify next-generation G-quadruplex targeted ligands with improved treatments and reduced cytotoxicity.

## 8 Exploration of G-quadruplex structures-dietary flavonoid interactions as probable molecular targets for future disease managements

Quadruplexes are involved in a variety of biological activities, including the translation and transcription of many oncogenes and tumour suppressors, as well as telomere stability and genomic instabilities. In this respect, quadruplexes have sparked research into their potential significance in cancer biology as well as the assessment of small-molecule ligands as prospective therapeutic drugs [75,76]. Experiments combining computational, biochemical, cell biology and molecular methodologies have shown that quadruplexes are abundant in the human genomes. The use of computer techniques to look for quadruplex consensus sequences aided in identifying quadruplexes and revealing their richness.

Dietary flavonoid's ability to act as anti-proliferative and anti-tumorigenic agents has earlier been documented but is not completely comprehended. One of the methods through which these flavonoids exhibit anticancer activities might be by targeting human telomeric G-quadruplex DNA. According to scientific investigations, a flavonoid-rich diet is associated with a lower risk of breast cancer. Although the potential relevance of nutritional and dietary aspects in cancer treatment, and diagnosis has been demonstrated [77,78], the need for further research into the diet-cancer association remains apparent. The exorbitant expense of regular cancer therapies, as well as the failure of most standard treatments, has prompted the healthcare professionals to seek out cost-effective preventative and therapy options. As a result, natural substances have received a lot of attention in the cancer sector. The biological ability of quercetin may be increased using nanostructured techniques, and a synergistic impact is shown when it is combined with other medications. In an article, this has been addressed that the use of natural chemical nutrition as a supplementary cancer medicines, dietary supplement, or adjuvant with anti-cancer compounds that might be used in conjunction with conventional breast cancer therapies, which requires more clinical research [79].

**Fig. 6:**
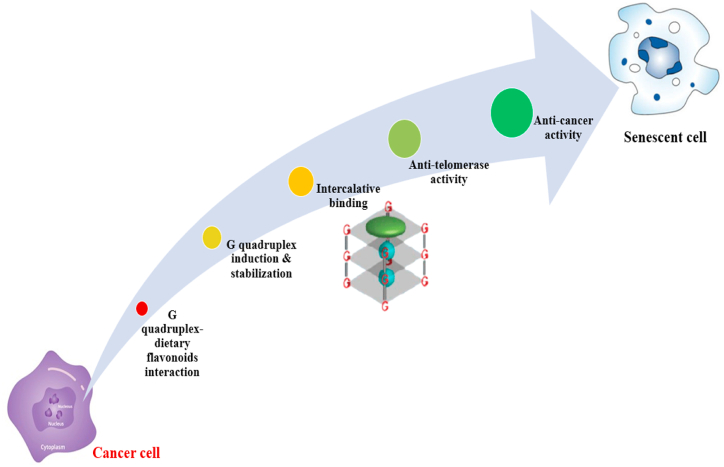
Schematic diagram of conversation from cancer cell to senescent cell by G-quadruplex-dietary flavonoid interaction.

Despite several investigations on these flavonoids’ anti-proliferative, anti-tumour and anti-apoptotic effects, the primary cellular mechanism of their activity remains unknown. Furthermore, it has been demonstrated that ligands containing planar aromatic domains intercalate and stabilise the G-quadruplex structure. The flavonoid skeleton has a planar chromophore with an extra carboxyl group for charge transfer and may successfully intercalate into the G-tetrad planar scaffold. Previous study focuses on the structural characteristics of flavonoid attachment to G-quadruplex structures generated by the telomeric DNA sequence of human, as well as the possibilities of flavonoids as anti-cancer treatments by modulating the telomeric G-quadruplex organization [65]. Dietary flavonoids can bind with DNA in a selective or non-specific manner, affect DNA activity to accomplish therapeutic or disease preventive targets, and also have multi-modal impacts with minimal toxicity. As a result, people are paying increasing attention to dietary flavonoids in cancer therapy and prevention. A schematic diagram represented in Fig. 6 that how a cancerous cell may convert into senescent cell by G-quadruplex-dietary flavonoid interaction. Nowadays, computer simulation and spectroscopy are mostly used to investigate the biophysical parameters (for example, the interaction pattern) of flavonoids and DNA associations [80]. The effects of cell proliferation management and apoptosis stimulation on cell signalling pathways have also been uncovered. Nonetheless, full knowledge based on interconnected biophysical, biochemical, and physiological data is still desperately required. To solve this issue, a thorough research of the interaction of dietary flavonoids with DNA at various levels is necessary.

Despite the fact that there is still a long way to go before the production of powerful drugs, some possible lead substances have been developed; nonetheless, the outcomes have been restricted thus far. To begin, the diversity of binding locations for these ligands, as well as the variation in their impacts on quadruplex structures, start making it difficult to determine how quadruplexes affect biological functionality, i.e., whether quadruplex destabilisation or stabilisation helps promote or hinders gene expression. Second, the relationship between *in vitro* stability and cell activities is not apparent. A G-quadruplex target identified *in vitro*, for example, might not be the only G-quadruplex directed in cells. In addition, there is intrinsic cell variability that influences the correlation between *in vivo* and *in vitro* observations. Another issue for the bulk of the ligands identified so far is that they have higher molecular mass and protonated side groups that might impact their cell absorption. Furthermore, the main barrier to practical use of quadruplex ligands appears to be connected to selectivity. The selectivity patterns of some quadruplex ligands are dose dependent. While universal or numerous G-quadruplex targeting strategies may be useful, targets must be explicitly identified ahead of time. Other possible impediments include the ligands' potential adverse effects on normal tissues. Furthermore, predicted response indicators must be established in order to provide customised anticancer treatment. Nonetheless, given the enormous collection of data on quadruplex architectures and the biological roles associated with them, as well as the fast evolution of ligands, we are hopeful that these restrictions may be addressed. A plethora of novel compounds with lesser cytotoxicity and higher selectivity will evolve in the near future in this approach.

The molecular influence of G-quadruplex structures-flavonoids interaction on essential cancer processes is anticipated to offer up new pathways in cancer treatment and detection. In this respect, the interaction of two specific regions is critical: (A) G-quadruplex structures generated in cancerous cells that can serve as possible molecular targets; and (B) small compounds capable of binding, stabilising, and perhaps visualising G-quadruplex complexes. Investigating the interactions and mechanisms of flavonoids with G-quadruplex DNA would aid in the development of appropriate dietary treatments and adjuvant medications for cancer therapy, which is critical to medical and people's health. To describe the mechanism of action by which dietary flavonoids are interacted with G-quadruplexes, more research at the proteome and genome levels is required. Insights into the processes by which dietary flavonoids affect cellular activities may be gained via mapping the direct interactions of flavonoids with G-quadruplex DNA. This work may also open the door for understanding, forecasting, and managing flavonoid responses in human.

## 9 Conclusion & future perspectives

The present review compiles information from the literatures and from the most recent studies and then compares the interactions of various flavonoids with their targets and concentrating on the links between dietary flavonoid structure and activity with G quadruplexes. Additionally, the various approaches for assessing interactions between dietary flavonoids and G-quadruplex DNA are highlighted. Natural substances have traditionally been useful companions in the fight against many diseases. The search for quadruplex-specific ligands has progressed from the early problem of identifying quadruplexes from canonical DNA to the current challenge of differentiating quadruplex structures from each other. Flavonoids are reported to influence a diverse panel of carcinogenesis processes, making them promising candidates for both tumor prevention and therapy. Flavonoids, in fact, were shown to control apoptosis as well as inhibit proliferation and migration, both of which are important processes in the development of cancer. Since G-quadruplex-DNA structures have evolved as a unique class of molecular target for anticancer medications, it is critical to examine correlations between dietary flavonoids and G-quadruplex-DNA in the search for G-quadruplex-DNA targeting therapies. Overall, these flavonoids have promising potential, and further research is needed to properly define its pharmaco-toxicological characteristics and analyse their prospective utility in chemoprevention and complementary therapy regimens. This was concluded that G-quadruplex-DNA-flavonoids interactions are a viable target for therapeutic development and that additional pharmacology research using *ex vivo*, *in vivo* and *in vitro* models is recommended. The application of this integrative research methodology provides a prospective avenue for the development of novel flavonoid-based phytomedicines. Several research suggests that there is no universal rule that describes the interactions between ligands and G-quadruplex-DNA, and that a sequence-dependent interaction may be essential as well. The role of the flavonoids' structure and substitution patterns on their affinities and binding modes to their target DNAs can be precisely assessed through the complementary application of additional experimental biophysical strategies and theoretical (molecular modelling) investigations. Further research into the mechanics of interactions between dietary flavonoids with G-quadruplex structures will be necessary in the future. Finally, G quadruplex-dietary flavonoid interactions will pave the way for the next generation of biology and revolutionary molecular medicine.

## Author contribution statement

All authors listed have significantly contributed to the development and the writing of this article.

## Funding statement

Sagar Bag was supported by University Grants Commission, India,{201610001623}, Mangal Deep Burman was supported by 10.13039/501100001412CSIR, India, {09/028(1154)/2020-EMR-I}, Dr. Sudipta Bhowmik was supported by SBV Intramural Seed Money Research Committee, {SBV/IRC/10.13039/501100001845SEED MONEY/134/2022}

## Data availability statement

Data included in article/supp. Material/referenced in article.

## Declaration of competing interest

The authors declare that they have no known competing financial interests or personal relationships that could have appeared to influence the work reported in this paper.
